# Editorial: Plant ER Stress and the UPR Signaling Pathways

**DOI:** 10.3389/fpls.2022.968353

**Published:** 2022-06-28

**Authors:** Lingrui Zhang, Diane C. Bassham, Barry R. Pittendrigh

**Affiliations:** ^1^Department of Entomology, Purdue University, West Lafayette, IN, United States; ^2^Department of Genetics, Development and Cell Biology, Iowa State University, Ames, IA, United States

**Keywords:** ER stress, UPR signaling pathway, unconventional splicing, abiotic stress, biotic stress, misfolded proteins

Plants are constantly subjected to various abiotic stress factors and biotic challenges, and have evolved highly complex and sophisticated adaptation mechanisms to cope with these adverse environmental stresses. One mechanism senses endoplasmic reticulum (ER) stress, in which the assigned capacity of the ER for *de novo* folding or refolding of proteins with high fidelity is perturbed. As a result, unfolded or misfolded proteins accumulate in the ER lumen. ER stress triggers an evolutionarily conserved signaling pathway designated as the unfolded protein response (UPR), such that stress circumstances in the ER are transmitted back into the nucleus to facilitate the expression of the UPR molecular signature genes, either functioning as an attempt to restore ER homeostasis or promoting cell death under unresolvable ER stress conditions.

As the importance of ER stress and UPR signaling in abiotic and biotic stress become increasingly recognized, establishing a diagnostic method to efficiently monitor UPR activation and dissect the function of various variants of ER stress transducers rapidly is becoming an urgent need. In an original method paper for this Research Topic, Diwan et al. developed a robust protocol for quantitative *bZIP60* mRNA substrate cleavage mediated by the ER transducer IRE1a, which is equipped with dual protein kinase and ribonuclease (RNase) activities. Besides demonstrating the essential character of a conserved amino acid in its RNase domain for mRNA substrate cleavage, the authors also demonstrate that the substitution of two amino acids in the kinase domain of IRE1a directly influences the function of the RNase domain. This contribution provides a platform for quickly determining the effects of IRE1 mutations on mRNA substrate cleavage activity and, therefore, guidance for the precise editing of the IRE1 transducer with the assistance of the CRISPR/Cas9 system by stable transformation *in planta*. Accordingly, researchers can utilize this method to test the compatibility of the mRNA-enzyme duet by employing variants of the stem-loop, which is embedded in the mRNA substrate and crucial for IRE1 recognition and cleavage. With minor modifications, this platform can be extended to investigate IRE1 interactors, such as BiPs or misfolded proteins, and screening chemical libraries for inhibitors or activators of IRE1. Considering that the mechanisms underlying IRE1 activation and regulation remain largely unknown *in planta*, this platform might be a valuable aid for illustrating how IRE1s sense stresses, undergo conformational changes, and transduce signals from the ER to the nuclei.

Plant growth and development are multifactorial events regulated by a complex and interactive network of regulators that integrate internal and external signals. Light is the first significant signal in plant growth and development and is integrated across various cellular pathways to complete a full life cycle. ELONGATED HYPOCOTYL 5 (HY5) has been demonstrated to be a negative regulator for mediating crosstalk between light signaling and the UPR (Nawkar et al., 2017). Original research in this Research Topic by Ahn et al. further takes us to the role of phytochrome B (phyB) in integrating light signaling and the UPR pathways to drive and adapt plant growth. They provide evidence that under ER stress, ER stress response genes, UPR-related bZIP transcription factors, and programmed cell death (PCD) associated genes were upregulated in *phyB*-overexpressing plants, but not in *phyB-5* mutants. These results demonstrate phyB as a positive regulator for integrating light signaling with the UPR to relieve ER stress and maintain proper plant growth. The findings also highlight that the N-terminal domain of phyB is essential for signal transduction of the ER stress response to the nucleus, which allies with light signaling.

Audiences interested in the molecular mechanisms that elicit PCD and autophagy signaling due to ER stress will find an updated and comprehensive summary of how plants deal with chronically prolonged ER stress in the review by Simoni et al. In this work, a detailed comparison illustrates the signaling of all UPR branches and describes the IRE1-mediated unconventional splicing and resultant spliced product of *HAC1, XBP1, AtbZIP60, GmbZIP68*, and *OsbZIP74* in the three kingdoms of *Animalia, Fungi*, and *Plantae*. The authors also highlight and clarify three signaling pathways for ER-induced PCD. Firstly, bZIP28 and bZIP60 matured from posttranslational modification or unconventional splicing can upregulate pro-apoptosis genes. The second plant-specific arm of ER-induced PCD is the developmental cell death (DCD) domain-containing, asparagine-rich protein (NRP)-mediated cell death response. Prolonged ER stress will also promote calcium accumulation and reactive oxygen species (ROS) burst in the mitochondria, leading to the activation of typical apoptosis pathways. The authors also emphasize recent progress on ER stress-induced PCD in plant immunity responses and ER stress-mediated autophagy, which is triggered to degrade some of the misfolded/unfolded proteins accumulated in the ER upon continuous ER stress.

Two exciting articles in this Research Topic (a review by Vitale and Pedrazzini and original research by Lohani et al.) focus on the UPR in the specialized plant reproductive unit. The former provides an in-depth and comprehensive summary of the current knowledge on the specific UPR during seed development. The accumulation of very high amounts of storage proteins and even selective degradation of specific storage proteins soon after their synthesis in seed cells introduces a significant challenge to the ER machinery. However, as pointed out by Vitale and Pedrazzini, the underlying signaling details remain largely unknown and present a key Research Topic area for the future. In the original research by Lohani et al., novel experimental data obtained by transcriptomic sequencing in *Brassica napus* anthers exposed to heat stress reveal a rapid transcriptional reprogramming mainly associated with the UPR. This finding suggests an activation of the UPR as an immediately responding critical pathway of heat stress response in gametophyte development. The authors also demonstrate that microspores are the primary site for expressing the rapidly responding genes; accordingly, they advocate for multi-omics studies to unravel complex cellular responses during reproductive development in response to stresses.

## Concluding Remarks

Collectively, this special topic highlights the pivotal role of ER stress and UPR signaling pathways in plant growth and development and stress responses. Some of the most recent advances summarized in the review articles, the establishment of a platform for evaluating ER sensors' function, and attempts to unravel UPR signaling in the plant reproductive unit selected for this Research Topic can be of inspiration for further work in this field. We envisage that these and future advances in this field will pave a path for the genetic control of the UPR signaling pathways to adapt plant growth and development.

## Author Contributions

LZ wrote the editorial, while DB and BP revised the text providing intellectual contributions. All authors contributed to the work and approved it for publication.

## Funding

Work in DB's laboratory was funded by grant # MCB-2040582 from the National Science Foundation.

## Conflict of Interest

The authors declare that the research was conducted in the absence of any commercial or financial relationships that could be construed as a potential conflict of interest.

## Publisher's Note

All claims expressed in this article are solely those of the author and do not necessarily represent those of their affiliated organizations, or those of the publisher, the editors and the reviewers. Any product that may be evaluated in this article, or claim that may be made by its manufacturer, is not guaranteed or endorsed by the publisher.
